# Follow-up non-attendance after long-bone fractures in a low-resource setting: a prospective study of predictors and interventions to increase attendance rates

**DOI:** 10.1186/s12913-023-10453-3

**Published:** 2023-12-13

**Authors:** Stephen Adesope Adesina, Isaac Olusayo Amole, Akinsola Idowu Akinwumi, Adepeju Olatayo Adegoke, Adewumi Ojeniyi Durodola, James Idowu Owolabi, Olufemi Timothy Awotunde, Imri Goodness Adefokun, Simeon Ayorinde Ojo, Samuel Uwale Eyesan

**Affiliations:** 1 Bowen University Teaching Hospital, P. O. Box 15, Ogbomoso, Oyo State Nigeria; 2 Bowen University, P.M.B 284, Iwo, Osun State Nigeria; 3 Adeleke University, P.M.B 250, Ede, Osun State Nigeria

**Keywords:** Follow-up, Non-attendance, Low-resource setting, Locked intramedullary nail, Long-bone fracture, Low- and middle-income countries

## Abstract

**Background:**

While the majority of traumatic injuries occur in low- and middle-income countries, the published literature comes chiefly from high-income countries due to poor follow-up. Clinical and radiographic post-surgical trauma follow-up is essential to high-quality research and objective monitoring for healing and/or complications. This study aimed to identify the predictors of follow-up non-attendance in a low-resource setting and investigate the extent to which interventional efforts based on mobile phone technology (MPT) and home visits improved the follow-up rates for fractures treated with SIGN nails.

**Methods:**

This was a prospective study of 594 patients with long-bone fractures. Socio-demographic (e.g. age, gender, marital status, education level, etc.) and clinical (e.g. fracture type, concomitant injuries, comorbidity, etc.) data were collected on each patient. Before discharge, the importance of follow-up was explained to patients and their relations. They were encouraged to attend even if they felt well. Their residential addresses and telephone numbers were validated and securely stored. Patients who missed their appointments were contacted by phone. Those who failed to honour 2 or 3 rescheduled appointments were visited in their home. The patients were divided into those who returned for the primarily scheduled follow-up without prompting (*volition group*) and those who did not come (*non-attenders*). Univariate analyses and binary logistic regression were conducted to determine the significant predictors of non-attendance.

**Results:**

The proportion of patients in the *volition group* reduced from 96.1% at 6 weeks to 53.0% at 12 weeks and 39.2% at 6 months. However, interventional efforts increased these values to 98.5%, 92.5%, and 72.4% respectively. Walking unaided before the primarily scheduled 12-week appointment was the major reason for not attending the follow-up. Education, occupation, post-operative length of hospital stay (PLOS) and infection were significantly associated with non-attendance but younger age, long distances from the hospital, being separated or divorced, difficulty paying the in-patient care bill, closed fracture, having no (or a non-limb) concomitant injury, achieving painless weight bearing ≤ 6 weeks post-operatively and needing no additional surgery were independent predictors of non-attendance.

**Conclusions:**

Our study sheds light on the predictors of follow-up non-attendance and demonstrates how interventional efforts improved attendance rates in a low-resource setting. In addition, efforts that better the socio-economic status of people such as more-encompassing health insurance coverage and greater work flexibility can improve the follow-up attendance rates.

## Background

Traumatic injuries have now grown to an epidemic proportion worldwide, and road traffic injuries alone are envisaged to be the third largest contributor to the global burden of disease by 2030 [1]. The majority of these injuries occur in low- and middle-income countries (LMICs) where trauma accounts for a substantial burden in terms of death, disability, and cost. Despite this fact, the published trauma literature comes chiefly from high-income countries (HICs) and studies in LMICs represent only a minority [2]. Home-grown research works published in credible outlets are essential for governments and communities in LMICs to make informed decisions towards reducing trauma-related deaths and disabilities. Inadequate follow-up is however, one major challenge preventing researchers in developing countries from getting their studies published in highly-rated journals [3, 4].

Clinical and radiographic follow-up is essential to post-surgical fracture care since it allows for objective monitoring for healing and/or complications [5, 6]. Out-patient follow-up provides an important point of continued care to trauma patients after hospital discharge, and some orthopaedic patients who are lost to follow-up have worse outcomes than patients who continue to return for evaluation [7, 8]. Moreover, long-term trauma outcomes research requires adequate follow-up as in-hospital data alone are insufficient for such research [7]. Many highly-rated journals require long-term high follow-up rates, and loss of follow-up has been a recurrent hindrance to trauma researchers, even in HICs [4, 6]. A maximum of 20% loss of follow-up is commonly accepted for prospective randomized trials [9].

There is no “gold standard” timeline for adequate follow-up of fractures [10]. A recent survey of Orthopaedic Trauma Association members who practised in the United States suggested that most clinicians consider clinical and radiographic evaluation up to 6 months sufficient for the majority of fractures, except for intra-articular fractures for which follow-up beyond 6 months was routinely considered [4]. In LMICs where advantages such as higher literacy levels, health insurance and basic amenities that encourage better post-surgical follow-up in HICs are lacking, getting trauma patients to return for follow-up even over a shortened timeline remains a big challenge. Patients often self-stop further follow-up once they regain the use of their limbs to an extent that allows them to return to their pre-injury activities and work, irrespective of radiological findings or the doctor’s opinion [3, 11, 12]. In a study by Young et al. in Malawi, only 58% of 137 patients with femur fractures returned for follow-up as scheduled but the patients with complications returned without prompting [3].

Consequently, some previous authors opine that it is unrealistic to insist on very high follow-up rates in clinical research from developing countries, arguing that such can leave out important information from the literature [3]. This argument appears valid when patients’ excuses for not honouring follow-up appointments, such as the high cost of transport, lack of money for radiographs and the fact that they have no complaints, are considered [3, 12, 13]. Studies in HICs have similarly found that sociodemographic and clinical factors such as age, income level, health insurance status, level of education, distance from the hospital, mechanism of injury, type of treatment (operative/non-operative) and hospital length of stay influence follow-up attendance [7, 14–16]. There are disparities in the findings of these studies, attesting to the position of Norquist et al. [16] that the underlying characteristics that incline a patient towards follow-up non-attendance are difficult to identify and control.

While there are reasons for declining follow-up appointments and insistence on 100% follow-up for research in LMICs may be excessive, our experience with follow-up of long-bone fractures fixed with the Surgical Implant Generation Network (SIGN) locked intramedullary nail (LIMN) in a semi-urban city of Nigeria showed that higher follow-up rates are possible. The SIGN LIMN are supplied free of charge to hospitals in LMICs and a minimum of 30% follow-up rates at the 6th and 12th post-operative week are compulsory for continuous supply. Hence, hospitals that adopt the traditional passive follow-up model in which doctors schedule appointments and expect patients to return without prompting may forfeit further implant receipt. We, therefore, adopted a proactive model in which patients are actively encouraged to return for follow-up, and if they fail, reach out to them by phone or home visit. This model helped us beat the cut-off and pushed the attendance rates much higher.

We hypothesize that trauma doctors in LMICs can make the most of the increasing ownership of mobile phones and widening telecommunication coverage to obtain better post-operative follow-up figures as some medical disciplines are doing [17–19]. Yet, there is a dearth of previous studies that explored this model to achieve high orthopaedic follow-up rates in our environment. In the study by Young et al. [3], the follow-up rate was increased from 58 to 79% by a combination of phone calls, phone interviews, and outreach visits. However, while their study mentioned some reasons patients adduced for not attending, it lacked a statistical evaluation of the predictors of non-attendance. Obtaining information about predictors of non-attendance of follow-up can help trauma surgeons design and implement appropriate follow-up protocols specifically targeted at high-risk patients. Hence this study aimed to (1) identify the sociodemographic and clinical factors that predicted follow-up non-attendance, and (2) investigate the extent to which interventional efforts based on mobile phone technology (MPT) and home visits improved the follow-up rates for long-bone fractures treated with SIGN LIMN over 8 years.

## Methods

### Background of the study centre

The study was undertaken at a teaching hospital in a semi-urban city in southwestern Nigeria. The city is inhabited by artisans, civil servants, subsistence farmers and small business owners. The hospital served other nearby villages/towns composed of similar populations. However, since SIGN Fracture Care International started supplying implants to our centre, patronage for fracture care by patients living in distant cities has considerably increased. These populations were generally poor, with little or no social welfare infrastructure to help trauma victims defray the cost of care. The available health insurance provided only partial coverage for the few civil servants in the population.

### Follow-up protocols

Beginning from post-operative day one, patients were made to ambulate. They were discharged from the hospital starting from post-operative day 4 onwards as permitted by their conditions. A follow-up appointment was given. The routine follow-ups were done at least twice – the 6th and 12th post-operative weeks. If painless weight bearing (PWB) was not achieved or there was no radiological evidence of ongoing healing at the 12th week, a patient was expected to return in the 6th post-operative month. Before discharge, the importance of honouring the follow-up appointment was explicitly communicated with the patients and their relations. They were actively encouraged to attend even if they felt all was well with their injured limb. The correct residential address and active telephone numbers of the patients were taken, validated and securely stored. The contact details of any easily accessible relations or friends of each patient were also documented especially if the patient had no phone. Consent was obtained to call the patients or their relations/friends to remind them of missed follow-ups and reschedule them. Patients also consented to be visited in their community if they failed to respond to telephone reminders. At follow-up, the fractures were assessed radiographically and clinically with PWB, ability to squat and smile (S&S) or do arm abduction and external rotation (AAER).

Each patient was expected to return for follow-up without prompting. However, any patient who missed the appointment was contacted by phone to ascertain their reason for not attending and to reschedule the visit. Patients who were absent because of financial difficulty were promised a subsidy on the cost of radiographs if they eventually came. Some patients who found returning inconvenient because they lived in distant cities were asked to send their radiographs and S&S or AAER pictures to the lead author via WhatsApp (*’tele-follow-up’*). Those who failed to honour the rescheduled appointment 2 or 3 consecutive times were visited in their home. If seen, they were clinically assessed and further encouraged to return for follow-up so that radiographic healing could be assessed. Those who were not met in their home had their family members interviewed to judge the patient’s recovery. We contacted every non-attender by telephone, either directly or through family/friends.

### Study design and statistical analysis

This study was a prospective study involving 594 patients with 652 fractures treated between July 2014 and June 2022. All patients whose humerus, femur or tibia fractures were fixed with the SIGN LIMN were included while those treated by other methods were excluded. The study protocols were approved by the Institutional Research Ethics Committee. All patients aged ≥ 18 years and the parents (or legal guardians) of those < 18 years gave informed consent to be included in the study. All study procedures were conducted per ethical standards. Socio-demographic data collected included age, gender, marital status, education level, occupation, in-patient care bill payment (easy/difficult) and distance of residence from the hospital. Clinical data were fracture type (open/closed), concomitant injuries, comorbidity, time PWB was achieved, need for additional surgery, infection, post-operative length of hospital stay (PLOS) and time from fracture to surgery. Patients were categorised into two based on occupation: (1) self-employed, which included artisans, commercial drivers/riders, farmers and traders who must be present at work to earn income from manual work or sales, and (2) salary-earning employees and dependents. There were also two categories under in-patient care bill payment: (1) ‘*easy*’ – patients who paid bills promptly, and (2) ‘*difficult*’ – patients whose relation had to take time to source funds before paying bills.

**Fig. 1:**
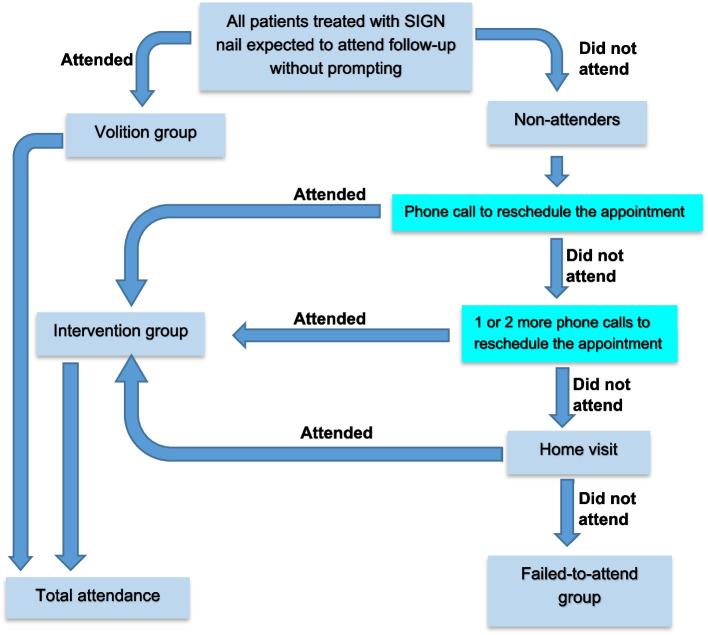
Flow chart depicting the follow-up cohorts

The data were analysed with SPSS version 23 (IBM Corp, New York, USA). As depicted in the flow chart (Fig. 1), the patients were divided into two broad groups: those who returned for the primarily scheduled follow-up without prompting (*volition group*) and those who did not honour the appointment and needed telephoning, home visit or *tele-follow-up* (*non-attenders*). The non-attenders were further divided into those who eventually attended (*intervention group*) and those who failed to attend despite all efforts (*failed-to-attend group*). Guided by the literature review and observation of patients’ behaviour following fracture surgeries in our setting over the years, we selected several variables to be tested as potential risk factors for non-attendance. Univariate analyses were performed on the selected variables in the 12-week follow-up data to determine the significant risk factors for non-attendance using Pearson’s chi-square (χ^2^) test for categorical variables and independent sample *t* test for numerical variables. The normality of the data was assessed visually with histograms to determine the appropriateness of parametric tests. Variables that demonstrated significance were then entered into binary logistic regression analysis as covariates to determine which were significant predictors of non-attendance. All *p* values were two-tailed, and the level of significance was set at *p* < 0.05.

## Results

**Fig. 2:**
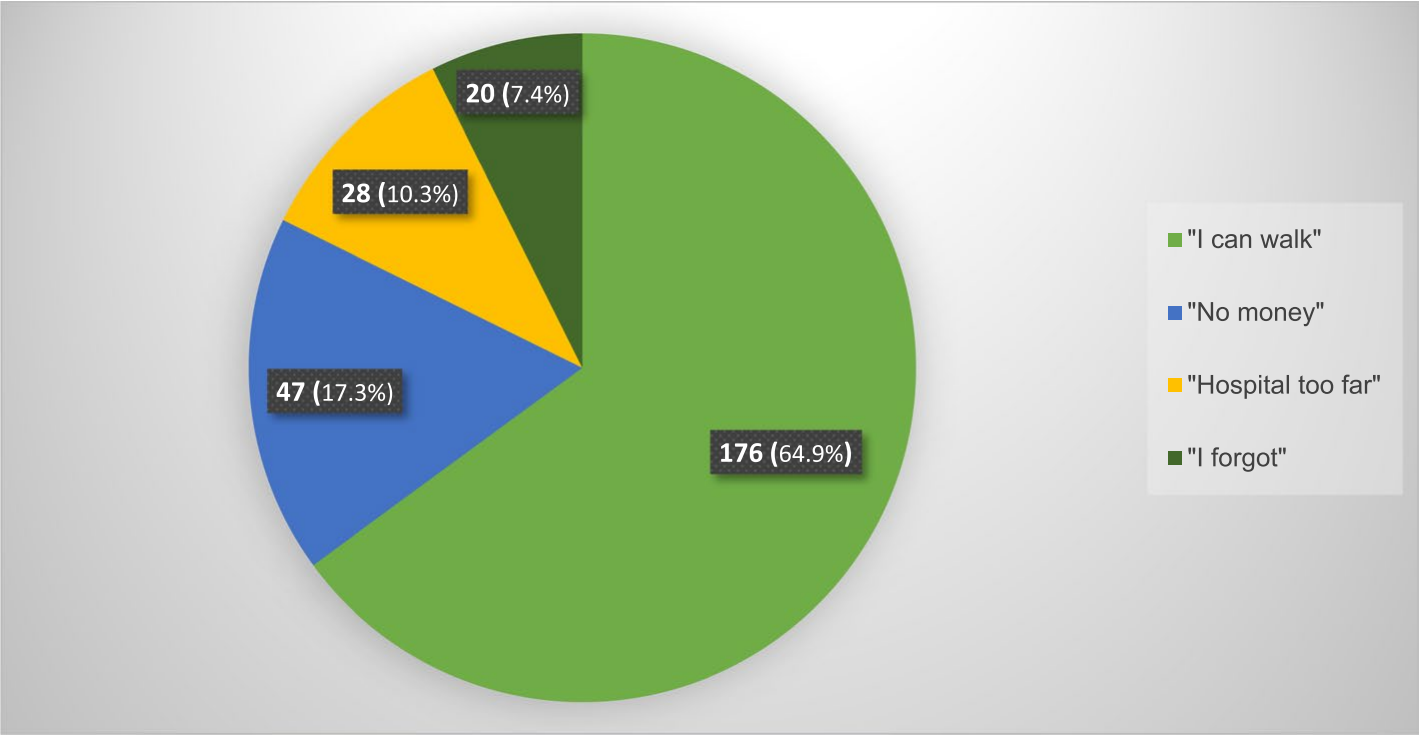
Reasons patients gave for non-attendance of the primarily scheduled 12-week follow-up appointment (n = 271)

The data displayed in Table 1 excluded the patients who died before their follow-up appointments. The proportion of patients in the *volition group* reduced as the postoperative time length increased, being 96.1% at 6 weeks, 53.0% at 12 weeks, and 39.2% at 6 months. However, interventional efforts increased these values to total attendance rates of 98.5%, 92.5%, and 72.4% respectively for the 6th-week, 12th-week, and 6th-month follow-up visits. The percentage of *failed-to-attend* increased with increasing postoperative time length, from 1.5% at 6 weeks to 7.5% at 12 weeks and 27.6% at 6 months. Figure 2 shows that patients who could walk unaided before the primarily-scheduled 12-week follow-up appointment constituted the majority of the non-attenders.

**Table 1:** Modes of follow-up attendance (*N* = 594)

**Follow-up attendance**	**6th-week (*n* = 584**^a^**)** **n(%)**	**12th-week (*n* = 577**^b^**)** **n(%)**	**6th-month (*n* = 301**^**c**^**)** **n(%)**
By volition	561 (96.1)	306 (53.0)	118 (39.2)
After interventional effort	14 (2.4)	228 (39.5)	100 (33.2)
Total attendance	575 (98.5)	534 (92.5)	218 (72.4)
Failed to attend	9 (1.5)	43 (7.5)	83 (27.6)

^a^Excluded 10 patients who died before the 6th-week follow-up

^b^Excluded 17 patients who died before the 12th-week follow-up

^c^Excluded 17 patients who died before the 6th-month follow-up and 276 who had healed and were not mandated to attend

In Table 2, comparisons using Pearson’s chi-square revealed that a significantly higher proportion of the Single and Separated/Divorced did not attend the primarily scheduled 12-week follow-up visit than the Married and Widowed (*p* < 0.001). Patients who had primary or secondary education were less likely to attend than those without formal education and the tertiary-educated patients (*p* < 0.001). Patients who were self-employed failed to come significantly (*p* = 0.028) more than salary earners and dependents. Patients with closed fractures attended less than those with open fractures (*p* = 0.015). Those who had concomitant injuries in the limbs attended more than those without a non-limb or no concomitant injury (*p* = 0.011). Difficulty with payment of in-patient care bills was significantly associated with follow-up non-attendance (*p* = 0.004). A higher proportion of patients who achieved PWB ≤ 6 weeks post-operatively than those who did after 6 weeks did not attend follow-up visits (*p* < 0.001). Patients whose fractures got infected attended significantly more than those without infection (*p* = 0.036). A need for additional surgery was associated with significantly less non-attendance (*p* = 0.001). Gender and presence of comorbidity did not have a statistically significant association with 12-week follow-up attendance.

**Table 2:** Categorical variables that influenced follow-up attendance at 12-week (*N* = 577^a^)

Variables		Volition (*n* = 306) n(%)	Non- attenders (*n* = 271) n(%)	Total	Test statistic (χ^2^)	*p*-value
*Sociodemographic variables*						
Gender	Male	197 (51.4)	186 (48.6)	383	1.166	0.280
	Female	109 (56.2)	85 (43.8)	194		
Marital status	Single	70 (45.8)	83 (54.2)	153	21.762	**0.000**
	Married	176 (54.5)	147 (45.5)	323		
	Separated/divorced	7 (25.9)	20 (74.1)	27		
	Widowed	53 (71.6)	21 (28.4)	74		
Education	None	53 (75.7)	17 (24.3)	70	18.890	**0.000**
	Primary	62 (47.0)	70 (53.0)	132		
	Secondary	101 (47.9)	110 (52.1)	211		
	Tertiary	90 (54.9)	74 (45.1)	164		
Occupation	Self-employed	155 (48.9)	162 (51.1)	317	4.834	**0.028**
	SE & Dp	151 (58.1)	109 (41.9)	260		
Bill payment	Easy	177 (58.8)	124 (41.2)	301	8.414	**0.004**
	Difficult	129 (46.7)	147 (53.3)	276		
*Clinical variables*						
Fracture type	Closed fracture	246 (50.8)	238 (49.2)	484	5.869	**0.015**
	Open fracture	60 (64.5)	33 (35.5)	93		
Concomitant injury	None	185 (50.1)	184 (49.9)	369	9.078	**0.011**
	Non-limb	59 (50.9)	57 (49.1)	116		
	Limb bone/joint	62 (67.4)	30 (32.6)	92		
Comorbidity	None/HTN	270 (53.4)	236 (46.6)	506	0.176	0.675
	Others	36 (50.7)	35 (49.3)	71		
PWB achieved ≤ 6 weeks	Yes	107 (38.9)	168 (61.1)	275	42.079	**0.000**
	No	199 (65.9)	103 (34.1)	302		
Infection	No	287 (52.1)	264 (47.9)	551	4.392	**0.036**
	Yes	19 (73.1)	7 (26.9)	26		
Additional surgery required	No	275 (51.2)	262 (48.8)	537	10.330	**0.001**
	Yes	31 (77.5)	9 (22.5)	40		

^a^Excluded the 17 patients who died before the 12th-week follow-up. χ^2^ = Pearson’s chi-square. *SE & Dp* Salary earners and dependents, *None/HTN* No comorbidity/controlled hypertension, *PWB* painless weight bearing. Statistically significant *p* values are indicated in bold

Table 3 shows the mean age for the non-attenders was significantly lower than that of the patients who returned by volition (38.9 vs 46.9 years, *p* < 0.001). Non-attenders lived significantly farther away from the hospital than those in the volition group (51.7 km vs 39.0 km, *p* = 0.036). The PLOS was significantly shorter for the non-attenders than the volition group (6.4 vs 7.4 days, *p* = 0.033). The association of time to surgery with 12-week follow-up attendance was not significant.

**Table 3:** Numerical variables that influenced follow-up attendance at 12-week (*N* = 577^a^)

Variables	Volition (*n* = 306)		Non-attenders (*n* = 271)		Test statistic (t)	*p-*value
	Mean	SD	Mean	SD		
*Sociodemographic variables*						
Patent's age (years)	46.9	19.43	38.9	15.82	5.444	**0.000**
Distance from the hospital (km)	39.0	55.28	51.7	84.22	-2.106	**0.036**
*Clinical variables*						
PLOS (days)	7.4	7.56	6.4	3.88	2.142	**0.033**
Time to surgery (days)	171.4	578.67	251.8	796.51	-1.373	0.171

^a^Excluded the 17 patients who died before the 12th-week follow-up. *SD* standard deviation, *t* Independent sample t-test. Statistically significant *p* values are indicated in bold. PLOS = postoperative length of stay

Table 4 displays the result of binary logistic regression to determine the independent predictors of non-attendance. The overall model was statistically significant when compared to the null model, χ^2^(17) = 112.4, *p* < 0.001, explained 23.6% of the variance in non-attendance (Nagelkerke R^2^), and correctly classified 68.5% of cases. Patients’ age (*p* = 0.014), distance from the hospital (*p* = 0.003), marital Status (*p* = 0.029), fracture type (*p* = 0.037), concomitant injury (*p* = 0.008), ease of bill payment (*p* = 0.002), PWB at ≤ 6 weeks (*p* < 0.001), and need for additional surgery (*p* = 0.008) were significant predictors of non-attendance but PLOS (*p* = 0.744), level of education (*p* = 0.341), occupation (*p* = 0.276) and infection (*p* = 0.440) were not.

**Table 4:** Binary logistic regression with non-attendance of the primarily scheduled 12-week follow-up as the outcome variable

Predictor variables (N = 577^a^)	B	aOR	*p*-value	95% CI	
				**Lower**	**Upper**
Patent's age (years)	-0.021	0.98	**0.014**	0.963	0.996
Distance from the hospital (km)	0.004	1.00	**0.003**	1.001	1.007
PLOS	-0.006	0.99	0.744	0.962	1.028
Marital status			**0.029**		
Single vs Widowed	-0.335	0.72	0.501	0.270	1.896
Married vs Widowed	-0.120	0.89	0.763	0.407	1.934
Separated/Divorced vs Widowed	1.244	3.47	**0.037**	1.075	11.192
Education			0.341		
Primary vs None	0.480	1.62	0.272	0.687	3.800
Secondary vs None	0.348	1.42	0.410	0.619	3.244
Tertiary vs None	0.715	2.04	0.108	0.856	4.883
Occupation	0.257	1.29	0.276	0.814	2.054
Fracture type	0.554	1.74	**0.037**	1.035	2.926
Concomitant injury			**0.008**		
None vs Limb bone/joint	0.850	2.34	**0.002**	1.355	4.042
Non-limb vs Limb bone/joint	0.799	2.22	**0.013**	1.184	4.177
Bill payment	0.649	1.91	**0.002**	1.279	2.860
PWB achieved ≤ 6 weeks	0.905	2.47	**0.000**	1.654	3.691
Infection	-0.547	0.58	0.440	0.144	2.317
Additional surgery required	1.575	4.83	**0.008**	1.495	15.606

^a^Excluded the 17 patients who died before the 12-week follow-up. *aOR* Adjusted odd ratio, *PLOS* postoperative length of stay, *PWB* painless weight bearing. Statistically significant *p* values are indicated in bold

For every one-year increase in age, the odds of non-attendance decreased by 0.98 (95% CI = 0.963 – 0.996). For every one-kilometre increase in a patient’s distance from the hospital, the odds of non-attendance increased by 1.00 (95% CI = 1.001 – 1.007). The odds of non-attendance for the Separated/Divorced were 3.47 times the odds for the Widowed (*p* = 0.037, 95% CI = 1.075 – 11.192). Patients with closed fractures were 1.74 times more unlikely to attend than those with open fractures (95% CI = 1.035 – 2.926). The odds of non-attendance for patients without a concomitant injury were 2.34 times the odds for those with limb bone/joint concomitant injuries (*p* = 0.002, 95% CI = 1.335 – 4.042) and 2.22 times the odds for those with non-limb injuries (*p* = 0.013, 95% CI = 1.184 – 4.177). Patients who had difficulty paying their in-patient care bill were 1.91 times more unlikely to attend follow-up than those who easily paid (95% CI = 1.279 – 2.860). The odds of non-attendance for patients who achieved PWB ≤ 6 weeks post-operatively were 2.47 times the odds for those who did after 6 weeks (95% CI = 1.654 – 3.691). Patients who required no additional surgery were 4.83 times more unlikely to attend than those who needed additional surgeries (95% CI = 1.145 – 15.606).

## Discussion

The findings from our study align with the observations of previous authors that follow-up non-attendance is a common problem in low-resource settings [3, 13, 20]. Despite our communication of the importance of keeping follow-up appointments with the patients before discharge, the attendance rate of the primarily scheduled follow-up appointments (*volition*) still reduced from 96.1% at 6 weeks to 53.0% at 12 weeks and 39.2% at 6 months (Table 1). However, interventions in the forms of phone calls and/or home visits increased these figures to total attendance rates of 98.5%, 92.5%, and 72.4% respectively, emphasising the need for proactive attendance-boosting efforts. Thus, pre-discharge education of patients on the importance of follow-up would be inadequate to make them return unless it is augmented by unrelenting post-discharge reminders and encouragement to honour the appointments.

Of particular note is the very high 6-week follow-up attendance by volition. Most of our patients were discharged home on walking aids. They needed the doctor’s judgement on when to discontinue the aids. Because the 6th-week visit was the earliest post-discharge appointment with their doctor, it may not be surprising that almost all of them attended this visit of their own accord. Since the LIMN provides stable fracture fixation and tolerates early WB [12, 21], most of our patients got the approval to start walking unaided at the 6th-week visit. Although this approval is scientific [22], many patients in our setting stop keeping follow-up appointments at this juncture irrespective of radiological findings. This is due to the belief that a fracture has healed once a satisfactory use of the injured limbs at work and other routine activities is achieved [11, 12, 23]. Interestingly, patients who did not return for the primarily scheduled 6th-week visit were found to be those allowed immediate WB without walking aids and had returned to work before the 6th post-operative week. Consequently, it became imperative to contact more patients by phone or visit to encourage them to return for further follow-up beyond the 6th-week visit.

Although its independent effect on follow-up attendance was not specifically explored, MPT was the foremost interventional effort made to improve follow-up attendance in this study. The effectiveness of MPT for improving patients’ follow-up has been reported by orthopaedic/trauma researchers in HICs [24, 25]. On the contrary, its use in LMICs is not yet popular. Leversedge et al. [26] in a systematic review of 18 studies on patient follow-up after orthopaedic outreach trips to LMICs, only three reported follow-ups via phone call or short message service (SMS). However, MPT has been widely used successfully in other disciplines [18, 27, 28]. In a randomized controlled trial in Nigeria, Thomas et al.found that individuals with first-episode psychosis who received SMS reminders were almost twice as likely to attend their appointment as the control group [28]. Similarly, Olajubu et al. [18] reported that sending educational and reminder messages to mothers’ phones significantly improved the uptake of postnatal care. A systematic review of the effect of patient reminders in reducing missed appointments in medical settings which included 20 studies showed that 95% of the studies reported a positive effect of patient reminders on appointment rates, with an average of 41% reduction in missed appointment rates [29].

The need for relentless post-discharge reminders and encouragement to attend follow-up is further highlighted by the fact that phone calls and/or visits defeated the pretexts for non-attendance in many of our patients. Out of the 271 patients who did not attend the primarily scheduled 12-week appointment, only 35.1% gave plausible excuses (funds, distance, forgetfulness) for not attending while 64.9% did not return because they adjudged themselves to have healed since they could walk (Fig. 2). By reminding these initial non-attenders, during phone calls or visits, that the early use of their limb was due to the sturdy implant and not because the fracture had healed, we succeeded in getting most of them to attend the rescheduled follow-ups. While patients’ subjective assessment of recovery is important [30], the benefits of objective evaluation of healing by medical experts during follow-up cannot be over-emphasised for the patients and health services [24, 27, 30, 31]. Nevertheless, there appears to be some plausibility in our patients’ subjective assessment of healing as most of those who walked unaided at 6 weeks reported no complications in later follow-ups, questioning the value of routine 12-week appointments and suggesting that patient-activated follow-ups would better utilise limited health resources. However, further corroboratory studies may be necessary.

Notwithstanding the pre-discharge education and post-discharge attendance-boosting interventions, the attendance rate reduced as the post-operative time length increased, suggesting the existence of factors exerting a defaulting influence on our patients. Statistical analysis of our data identified several such factors which were sociodemographic or clinical (Tables 2 and 3). Education, occupation, PLOS and infection were significantly associated with non-attendance in univariate analysis but were not independent predictors when entered as covariates in logistic regression. On the other hand, the remaining eight covariates were independent predictors of non-attendance (Table 4). A long-term prospective trauma study previously reported that patients lost to follow-up were demographically and clinically different from those who attended [15]. Financial difficulties appear to be the most reasonable explanation for the relationships between the sociodemographic variables and follow-up non-attendance. Conversely, a less severe injury and an uncomplicated care course with consequent early return to work most plausibly explain the influence of the clinical variables.

Age was an independent predictor of non-attendance in our study which agrees with prior researchers’ findings [15, 32]. Due to high unemployment and underemployment rates in Nigeria [33], the majority of young people up to the third decade of life have not attained financial stability that can independently navigate a major orthopaedic injury. The preponderance of the younger population among the non-attenders may also reflect their general irresponsibility towards medical follow-up. Similarly, longer distances of patients’ residences from the hospital independently predicted follow-up non-attendance. This is intuitive in that travelling long distances to the hospital often translates to a higher cost and transport difficulty [34]. It may also mean patients did not return because they have sought follow-up care somewhere more proximal than where their surgery was done.

Furthermore, being separated/divorced is an economically disadvantaged status in our society. Equally, primary/secondary-educated people were mostly self-employed people who must be present at work to earn income. Thus, they often become poor when fracture incapacitates them [12]. This group of patients failed to attend possibly because of the reduced flexibility in missing work and poor understanding of the need for follow-up. This suggests that efforts that better the socio-economic status of people, such as more-encompassing health insurance coverage, greater work flexibility, higher education and stronger family bonds, can improve the follow-up attendance rates in low-resource settings, in addition to the aforementioned pre-discharge follow-up education and unrelenting post-discharge reminders. ten Berg and Ring [35] reported that non-attendance of follow-up after metacarpal fractures was independently predicted by single/divorced status, lack of insurance and unemployment. Murnaghan and Buckley [15] reported that non-attenders of follow-up for calcaneus fractures were more likely to be unskilled workers. Tejwani et al. [6] found that patients lost to follow-up after distal radius fractures were more likely to be low-income earners who had not surpassed secondary education.

Concerning the clinical variables favouring non-attendance, short PLOS, No infection, Closed fracture, No (or non-limb) concomitant injury, PWB ≤ 6 weeks post-operatively and No additional surgery all spotlight a less serious injury with a straightforward treatment course and consequent early recovery. Many previous authors have noted that those who attend follow-ups are more likely to have a more serious injury and vice versa [6, 15]. Our patients with more severe injuries took longer to regain their pre-injury ability to perform daily activities and therefore possibly took follow-up visits more seriously than those who recovered earlier.

## Limitations

We are not aware of any earlier study on the predictors of non-attendance of orthopaedic/trauma follow-up in Nigeria. However, some limitations of this study must be acknowledged. There was no randomisation in assigning the patients into *volition* and *non-attender* groups. Patients did not get pre-appointment telephone reminders and they were neither promised a subsidy on the cost of radiographs nor offered the option of *‘tele-follow-up’* until they had missed the primarily scheduled appointment. Thus, some patients who could have attended follow-up by volition if they had money, lived near the hospital or did not forget their appointment date (Fig. 2) were classified as non-attenders. The lack of a control group is another possible limitation. Notwithstanding these limitations, this study provides information that could serve as a basis for a future randomised controlled trial.

## Conclusion

Our study sheds light on the risk factors for follow-up non-attendance and the high-risk groups whom trauma researchers must diligently monitor to improve follow-up rates. It also provides some evidence for the common but largely anecdotal saying in developing countries that ‘patients are lost to follow-up because they are well’. Using MPT and occasional home visits helped increase our follow-up rates. These interventions offer prospects for addressing the erstwhile difficulties faced by developing countries in tracking patients for follow-up. In addition, efforts that better the socio-economic status of people, such as more-encompassing health insurance coverage and greater work flexibility can improve the follow-up attendance rates in low-resource settings.

## Data Availability

The datasets used and/or analysed during the current study are available from the corresponding author upon reasonable request.

## Abbreviations

AAER: Abduction and external rotation of the arm
aOR: Adjusted odd ratio
HICs: High-income countries
HTN: Hypertension
LIMN: Locked intramedullary nail
LMICs: Low- and middle-income countries
MPT: Mobile phone technology
PLOS: Post-operative length of hospital stay
PWB: Painless weight bearing
SIGN: Surgical implant generation network
SMS: Short message service
SPSS: Statistical package for the social sciences
S&S: Squat and smile
WB: Weight bearing
